# SLE and purine metabolizing ecto-enzymes

**DOI:** 10.1016/j.ebiom.2021.103688

**Published:** 2021-11-10

**Authors:** Bruce N. Cronstein

**Affiliations:** NYU Grossman School of Medicine, NYU-H+H Clinical and Translational Science Institute, New York, NY 10016

Systemic Lupus Erythematosus (SLE) is a heterogeneous autoimmune disease characterized by hyperactive immune responses leading to severe and potentially life-threatening organ damage. Recent work indicates that in many patients increased interferon-driven responses are the root of the increased immune responses and the first therapy directed against the Type I Interferon receptor was recently approved for the treatment of SLE (Anifrolumab [1,2]).

A genetic polymorphism in purine nucleotide phosphorylase (PNP), which catalyzes the hydrolysis of adenosine and inosine to adenine and hypoxanthine, respectively, is strongly associated with the risk for development of SLE in patients with high levels of Interferon [3]. Although initial studies suggest that this defect in the purine salvage pathway is associated with purine starvation leading to cell death it is also possible that diminished activity of this enzyme leads to alterations in endogenous adenosine production. Adenosine, a potent regulator of the immune system is produced at the cell surface by the progressive dephosphorylation of adenine nucleotides to adenosine by the actions of ecto-nucleotide dephosphorylase (CD39) and 5′ecto-nucleotidase (CD73). Indeed, antagonists of the anti-inflammatory and immunosuppressive adenosine A2A receptor and inhibitors of CD73, the enzyme responsible for the final step in production of adenosine at the cell surface, are currently targets of check point inhibitor therapy for the treatment of malignancies (cf [[4], [5], [6],^5^]). Interestingly, patients lacking ecto- 5′nucleotidase activity develop severe vascular calcification, often leading to ischemia, gangrene and amputation and, thus, it is interesting to speculate that, at least in some patients, abnormalities in adenosine production or response may contribute to the dramatic activity of the immune system in patients with SLE.

In this issue of EBiomedicine Hesse and colleagues report that, although CD73 expression remains relatively unchanged, the enzymatic activity of CD73 is markedly reduced on the surface of B cells from patients with SLE [6]. Consistent with this observation, the level of AMP generated by these cells is markedly increased and the adenosine level is significantly lower than in cultures of B cells from normal control individuals. This finding is remarkable in that it is the first report of a change in CD73 activity not associated with a decrease in expression of the enzyme on the surface.

The authors speculate that the reduction of adenosine in the extracellular milieu of the B cells of these patients would lead to increased activity of the immune system, a finding that could predispose to the development of SLE. Nonetheless, it is unclear whether diminished enzymatic activity contributes to the development of SLE or is a result of SLE disease activity. It is also tempting to speculate that since CD73 is GPI-linked, like cell surface complement-regulatory proteins, that secondary alterations in the plasma membrane could lead to altered expression on the cell surface with diminished enzymatic activity. It would also be interesting to know whether the reduced activity of CD73 is associated with increased disease activity or manifestation of disease (skin vs kidney for example).

Nonetheless, these questions aside, the authors have made an interesting finding that could suggest a complementary mechanism for the overactivity of SLE lymphocytes and the resultant autoimmunity. These findings complement earlier observations of the effect of changes in purine metabolism which offer a potential metabolic basis for SLE.

## Declaration of Competing Interest

Dr Cronstein is Chair of the Scientific Advisory Board and holds stock in Regenosine, LLC, a company that has licensed technology patented by his team and assigned to NYU Grossman School of Medicine.
